# Hand, Foot and Mouth Disease in Hong Kong: A Time-Series Analysis on Its Relationship with Weather

**DOI:** 10.1371/journal.pone.0161006

**Published:** 2016-08-17

**Authors:** Pin Wang, William B. Goggins, Emily Y. Y. Chan

**Affiliations:** 1 School of Public Health and Primary Care, Faculty of Medicine, The Chinese University of Hong Kong, Hong Kong, China; 2 Collaborating Centre for Oxford University and CUHK for Disaster and Medical Humanitarian Response, School of Public Health and Primary Care, Faculty of Medicine, The Chinese University of Hong Kong, Hong Kong, China; University of Vigo, SPAIN

## Abstract

**Background:**

Hand, foot and mouth disease (HFMD) is an emerging enterovirus-induced infectious disease for which the environmental risk factors promoting disease circulation remain inconclusive. This study aims to quantify the association of daily weather variation with hospitalizations for HFMD in Hong Kong, a subtropical city in China.

**Methods:**

A time series of daily counts of HFMD public hospital admissions from 2008 through 2011 in Hong Kong was regressed on daily mean temperature, relative humidity, wind speed, solar radiation and total rainfall, using a combination of negative binomial generalized additive models and distributed lag non-linear models, adjusting for trend, season, and day of week.

**Results:**

There was a positive association between temperature and HFMD, with increasing trends from 8 to 20°C and above 25°C with a plateau in between. A hockey-stick relationship of relative humidity with HFMD was found, with markedly increasing risks over 80%. Moderate rainfall and stronger wind and solar radiation were also found to be associated with more admissions.

**Conclusions:**

The present study provides quantitative evidence that short-term meteorological variations could be used as early indicators for potential HFMD outbreaks. Climate change is likely to lead to a substantial increase in severe HFMD cases in this subtropical city in the absence of further interventions.

## Introduction

Since its original identification in New Zealand in 1957 [1], hand, foot and mouth disease (HFMD) has been frequently reported worldwide [2–5], primarily affecting children aged 5 years and younger. Predominantly transmitted through person-to-person contact, respiratory droplets or contaminated objects, HFMD is most commonly caused by coxsackievirus A16 (CA16) and enterovirus 71 (EV71) [6]. However, other strains of non-polio enteroviruses have also been reported to be causative pathogens [7]. Clinical manifestations of HFMD includes fever, sore throat and mouth, small vesicles, ulcers and rashes on the skins and mucous membranes of the oral cavity. Most patients recover within 7–10 days without any clinical attention due to its typically mild and self-limiting nature. However, systemic complications, including aseptic meningitis, acute flaccid paralysis, brainstem encephalitis, pulmonary edema or hemorrhage, and acute heart failure, are not uncommon, especially when associated with EV71 infection [8].

Asian countries have seen an increasing number of HFMD epidemics in recent years. A total of 2,819,581 newly diagnosed HFMD cases, and 394 fatalities, were reported in China in 2014 [9], substantially more than 1,855,457 cases in 2013 and 2,198,442 in 2012 [10]. In addition, the largest ever outbreak of HFMD in Singapore, resulting in 29,686 cases and one death, was observed in 2008 [11]. Although there has been very few death case seen due to HFMD in Hong Kong, thousands of people were affected every year. For instance, 691 outbreaks were recorded in 2015 and a total of 4,194 persons were involved [12]. As of Jun 4^th^ in 2016, there have been 285 institutional outbreaks observed and 249 hospital admissions have been reported [12].

Global efforts are still ongoing to identify the mechanism facilitating the continuous HFMD outbreaks observed in recent years [2–5]. Evidence of the seasonality of HFMD has been found in studies from many locations [2, 3, 13–16]. For countries with relatively higher latitudes, such as Finland [13] and Japan [16], a single peak of HFMD has been seen, during the summer, and autumn months, respectively. However, two annual peaks have been observed in regions with subtropical and tropical climates including Taiwan, Hong Kong, Malaysia, Singapore and parts of mainland China [2, 3, 14, 15, 17].

A relationship between meteorological factors and the incidence of various infectious diseases has been frequently observed [18, 19], and there are several plausible mechanisms whereby these factors could affect the breeding, growth, and transmission of pathogens, as well as human behavior. Several time series studies have found associations between HFMD and weather, but the results have been inconsistent [15–17, 20–28]. For example, studies from Singapore, Hong Kong, Japan and Guangdong, Henan, Shandong and Beijing in China have reported a positive association between temperature and HFMD [15–17, 21, 22, 27, 28], whereas a negative association has also been found [24]. In addition, disparity in the strongest effect of temperature was revealed as well, with an inverse-U relationship by one study, with 23°C (74°F) as the temperature with the maximum morbidity [23], while another study from Southern China reported more HFMD cases were associated with both high and low same-day temperature [25]. Although reported associations with temperature have been inconsistent, a significant and positive relationship between relative humidity and HFMD has been consistently reported [16, 17, 22–27]. Other weather factors, such as precipitation [15, 20, 23, 26], wind speed [22, 23] and sunshine [27], have been investigated as well. Extreme high precipitation was found to be associated with higher HFMD incidence among children in Hefei, China [20], while a study from Singapore reported an inverse-U association between weekly cumulative rainfall and HFMD with a peak at 75mm [15]. In addition, more HFMD cases were found to be associated with higher wind speed [23] in a nationwide study from 342 locations in China and with less sunshine [27] in Rizhao, China.

Using a long time series from a large database of cases from all public hospitals in Hong Kong, the current study aimed to quantify the association of daily weather variations and hospitalizations for HFMD in Hong Kong, a subtropical Chinese city, using modern regression methods and controlling for multiple environmental factors, long-term trends, and seasonality.

## Methods

### Data Sources

A clinical database from Hong Kong public hospitals, covering roughly 83% of all the inpatient admissions in the city, with discharge diagnoses encoded using the International Classification of Diseases, 9^th^ Revision (ICD-9, code for HFMD: 074.3) from the Hong Kong Hospital Authority was used for abstraction of daily counts of HFMD hospitalizations during the period 2008–2011. Daily values of meteorological variables, including mean daily temperature, relative humidity, and wind speed, and total daily rainfall and global solar radiation, were obtained from the Hong Kong Observatory.

### Statistical Analysis

Time-series regression analyses were used to model daily HFMD cases as a function of meteorological variation in Hong Kong. Distributed lag non-linear models [29] and generalized additive models (GAMs) [30], with a negative binomial distribution assumed to account for over-dispersion, were constructed to assess potentially non-linear exposure-response dependencies and delayed effects simultaneously. Taking the incubation period of 3–7 days for HFMD into account, we defined the maximum lag for meteorological factors as 14 days. Partial autocorrelation plots were used to determine the necessary number of autoregressive terms in the models. To adjust for seasonality and long-term trend, thin plate regression splines with 6 and 4 degrees of freedom (*df*), respectively, were incorporated in the models. Day of week and public holidays were also adjusted as categorical variables in all models. The model family can be formulated as follows:

$$\begin{array}{l} \text{log}\left[E\left({\text{Y}}_{t}\right)\right]=\beta_{0}+\sum cb\left(\text{meteorological variables},\;df_{1},\text{ lag},\;df_{2}\right)\;+s\left(\text{DOY},\;df=6\right)\\ +\;s\left(\text{t},\;df=4\right)+factor\left(\text{DOW}\right)+factor\left(\text{holiday}\right)+\text{autoregressive terms} \end{array}$$

where *E*(Y_*t*_) is the expected daily count of HFMD on day t; *β*_*0*_ is the intercept; *cb()* represents a crossbasis function, modeling exposure-response and lag-response relationships simultaneously with their corresponding degrees of freedom; *s()* denotes a thin plate regression spline function; DOY represents day of year controlling for seasonality; DOW and holiday mean indicator variable for day of week and binary variable for public holiday. Rainfall was included in the model as a categorical variable, with no rainfall as reference, and indicator variables for rainfall greater than 0 mm and less than 90^th^ percentile (17.7 mm) as moderate precipitation, and over 90^th^ percentile as an extreme category. Different degrees of freedom (3–6) for the crossbasis functions and smoothing spline functions were also tested to assess model robustness. The goodness-of-fit among the models were compared and validated using the regression coefficient (*R*^2^) and Akaike’s information criterion (AIC).

Interaction between daily mean temperature and relative humidity was assessed for 5, 7, 10, 14 days’ cumulative lags, respectively. Effect modification by gender, and age group (0–2 years, 3–14 years, over 14 years) were evaluated with stratified analyses accordingly.

All results are reported as the relative risk (RR) of a chosen percentile value of certain meteorological variable with corresponding 95% confidence interval (CI), compared to its 25^th^ percentile. All statistical analyses were performed using the dlnm() [29] and gam() [30] packages in R software version 3.1.0. An ethics exemption from the Survey and Behavioural Research Ethics Committee at the Chinese University of Hong Kong was obtained since the current study didn’t involve survey or observation of human behavior. In addition, patient records were anonymized and de-identified (names and ID numbers removed) prior to our receiving the data. The data that were analyzed and reported in the paper are aggregated data (daily counts).

## Results

### Descriptive Statistics

**Fig 1:**
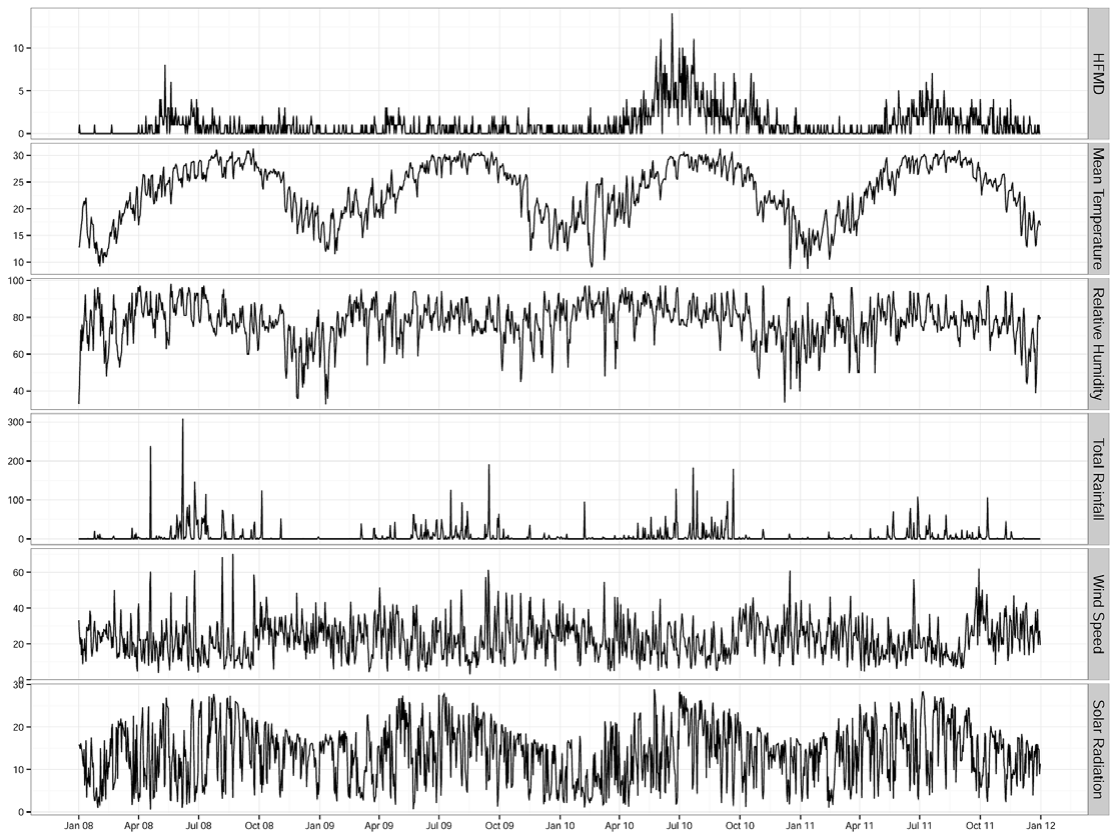
Seasonal variation in daily hand, foot and mouth disease cases and meteorological variables in Hong Kong, 2008–2011.

A total of 1,534 HFMD cases (585 females and 949 males) were hospitalized during the study period from Jan 1^st^ 2008 through Dec 31^st^ 2011 in Hong Kong, of which 984 (64.1%) were children aged 0–2 years, 455 (29.7%) were aged between 3–14 years, and 95 (6.2%) were aged over 14 years. Descriptive statistics for daily HFMD cases and meteorological conditions are summarized in Table 1. Fig 1 shows the time series of daily HFMD cases and all of the meteorological variables during the study period. A seasonal pattern was observed for HFMD, with a major peak in late spring (May–Jul) and a minor peak in autumn (Sep–Oct) for the years 2010 and 2011.

**Table 1:** Descriptive summary for hand, foot and mouth disease cases and meteorological variables in Hong Kong, 2008–2011.

	Mean	SD	Min.	P(25^th^)	Median	P(75^th^)	Max.
Daily HFMD	1.05	1.65	0	0	0	1	14
Sex							
Male	0.65	1.17	0	0	0	1	8
Female	0.40	0.78	0	0	0	1	7
Age							
0–2 years	0.67	1.12	0	0	0	1	7
3–14 years	0.31	0.71	0	0	0	0	8
>14 years	0.07	0.08	0	0	0	0	3
Temperature(°C)	23.25	5.31	8.80	19.1	24.40	27.9	31.20
Relative humidity(%)	77.51	10.86	33.00	73.00	78.00	85.00	98.00
Rainfall(mm)	6.22	20.45	0	0	0.01	1.40	307.10
Wind speed(km/h)	22.82	10.15	3.20	15.20	22.10	29.10	70.00
Solar radiation(MJ/m²)	13.85	6.67	0.64	8.30	14.42	18.54	28.81

### GAM Results

**Fig 2:**
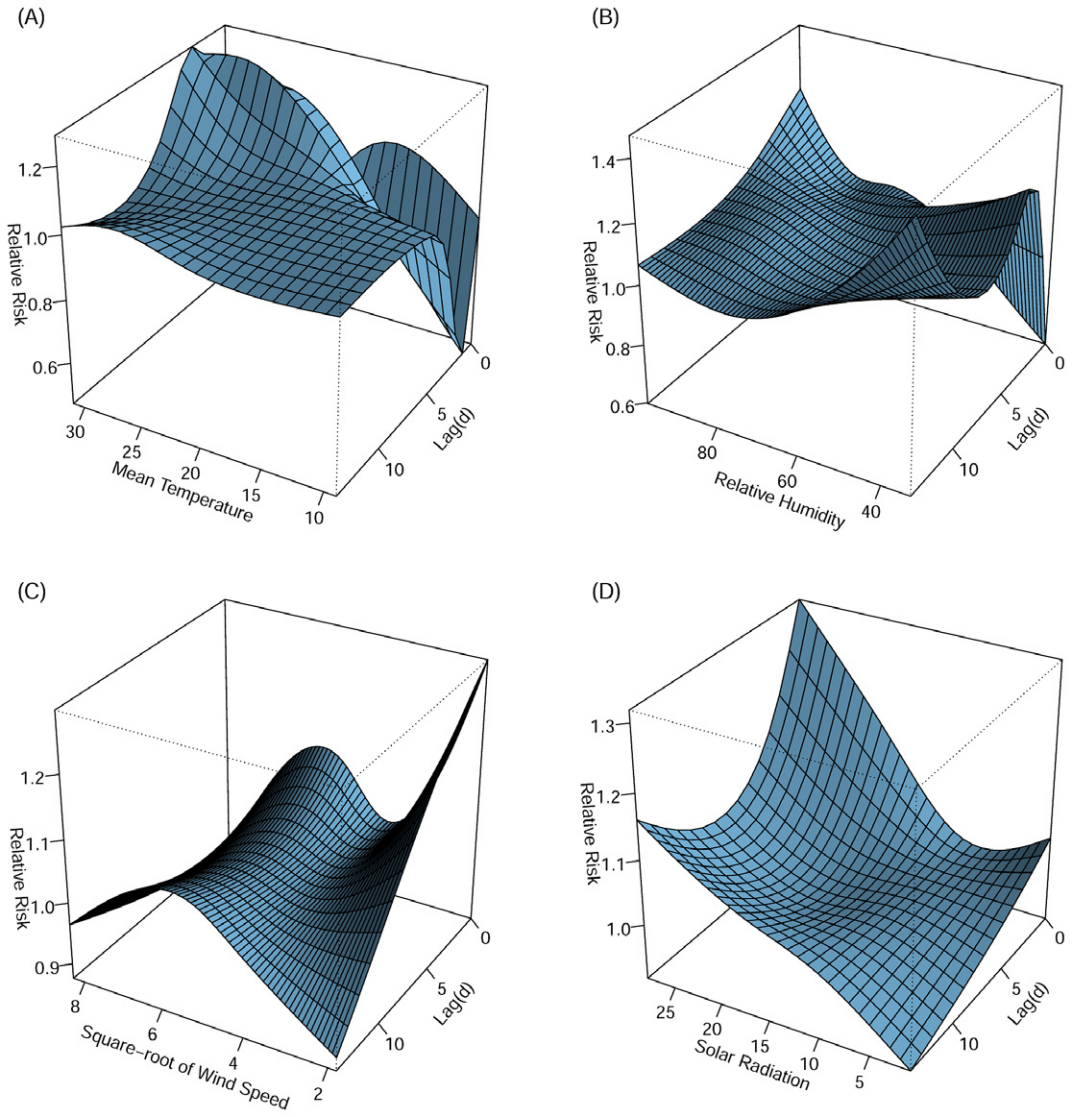
Three-dimensional plots of relative risks along daily mean temperature (A), relative humidity (B), square root of wind speed (C) and total daily solar radiation (D) and their corresponding lags.

Based on the partial autocorrelation function plots, autoregressive terms for lags 1–6 days for daily HFMD were included in the models. Fig 2 depicts the entire exposure-lag-response three-dimensional associations between meteorological variables and HFMD in Hong Kong, allowing for a maximum lag of 14 days. There was a strong positive association between temperature and HFMD at lag 1–3 days, followed by a weaker positive association at lag 4–7 days (Fig 2A). From the perspective of lag-response dimension, extreme temperature (over 30°C) were associated with highest relative risk for HFMD hospitalization at lag 1–3 days. A strong and immediate association of high relative humidity with HFMD, which weakened after 5–7 days, was seen (Fig 2B). The associations of wind speed and solar radiation were more consistent during the whole lag period. Same day associations between low wind velocity and high solar radiation with HFMD occurrence were also found (Fig 2C and 2D). Moderate wind speed was associated with HFMD hospitalizations with a peak at 36 km/h, with lagged effects lasting from the current day until 14 days later. Although generally decreasing with time, the positive association between HFMD and solar radiation persists during the entire 14 days’ lag time.

**Fig 3:**
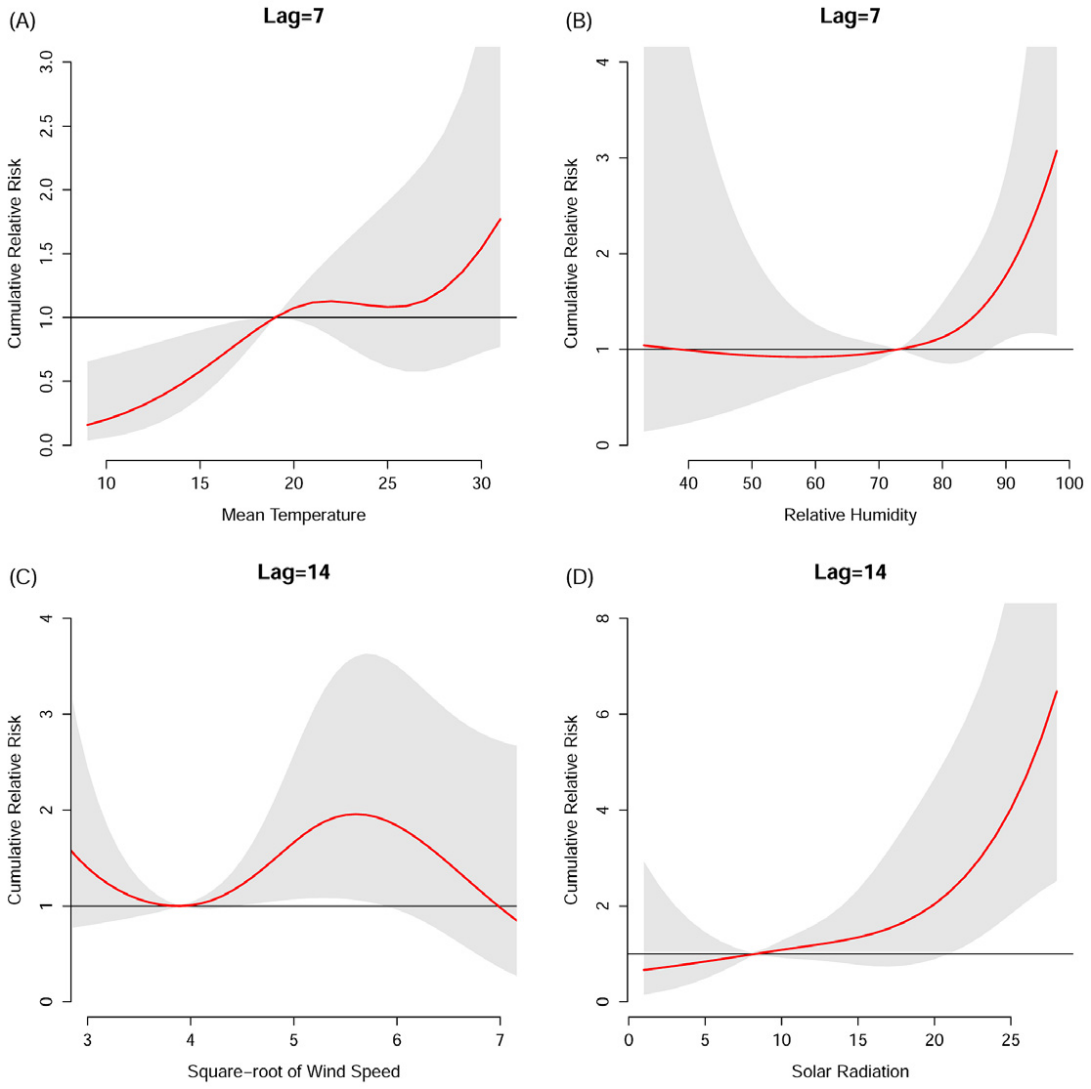
Cumulative relative risks of hand, foot and mouth disease hospitalization as a function of meteorological variables for a time lag of 1 week (mean temperature (A) and relative humidity (B)) and 2 weeks (square root of wind speed (C) and total solar radiation (D)).

**Fig 4:**
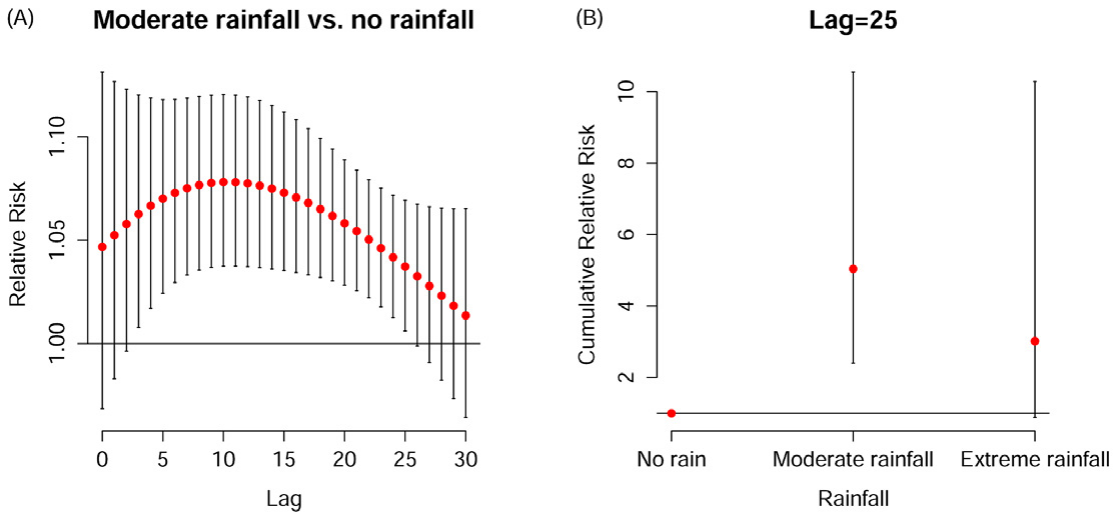
Lag-response association specific to rainfall category 2(0 mm<rainfall<17.7 mm) (A) and exposure-response cumulative association specific to lag 25 (B).

The corresponding cumulative relative risks, summing up all the contributions up to the chosen lag time, together with their 95% confidence intervals, are presented in (Fig 3A–3D). The lag days over which cumulative effects were summed were selected based on the prior three-dimensional plots. A positive relationship between mean temperature and HFMD was found from 8 to 20 degrees Celsius and above 25 degrees, although only the association over the former temperature range was significant (Fig 3A). A hockey-stick association was found for relative humidity with HFMD increasing significantly with rising RH over 80% (Fig 3B). Stronger wind (between 16 and 33.6 km/h) was significantly associated with more HFMD cases (Fig 3C). The association between solar radiation and HFMD was stronger for values > 15 MJ/m^2^ (Fig 3D). Fig 4 shows the lag-response association specific to moderate rainfall (Fig 4A) and the exposure-response cumulative association specific to lag 25 days (Fig 4B). We initially attempted 30 days of maximum lag to model its delayed effect, which could persist significantly until the 25^th^ day afterwards. Subsequently the cumulative relative risk on HFMD on the 25^th^ day was assessed. Moderate precipitation was associated with the highest risk of HFMD relative to both no rainfall and heavy rainfall, with a cumulative relative risk of 5.04 (95% CI 2.41–10.55) for moderate versus no precipitation. The cumulative effects of all the meteorological variables in the models are summarized in Table 2.

**Table 2:** Cumulative effects of different meteorological factors on hand, foot and mouth disease by different days of lag.

	5 days	7 days	10 days	14 days	25 days
Temperature (30 vs. 19°C)	1.47 (0.73,2.96)	1.54 (0.73,3.28)	1.40 (0.61,3.25)	1.42 (0.54,3.73)	N/A
Temperature (11 vs. 19°C)	0.25* (0.09,0.70)	0.25* (0.09,0.73)	0.26* (0.08,0.81)	0.26* (0.07,0.93)	N/A
Relative humidity (96 vs. 73%)	2.29* (1.13,4.63)	2.66* (1.17,6.07)	3.19* (1.17,8.72)	3.97* (1.14,13.78)	N/A
Wind speed (29.1 vs. 15.2 km/h)	1.36 (0.97,1.93)	1.43 (0.95,2.14)	1.56 (0.96,2.55)	1.95* (1.08,3.53)	N/A
Solar radiation (25 vs. 8.3 MJ/m^2^)	2.21* (1.40,3.47)	2.40* (1.42,4.03)	2.76* (1.48,5.15)	4.03* (1.83,8.83)	N/A
Rainfall (Moderate^a^ vs. no rain)	1.41* (1.01,1.99)	1.63* (1.08,2.45)	2.04* (1.25,3.33)	2.74* (1.53,4.91)	5.04* (2.41,10.55)

*Significant results

^a^Moderate rainfall refers to rainfall that is less than 17.7 mm (90^th^ percentile).

No significant interaction between mean temperature and relative humidity was found within 5,7,10 or 14 days’ time frame. The results of stratified analysis based on different gender, and age groups are presented in Table 3. The relative risk showed the cumulative risk of having a patient hospitalized due to HFMD on the 14^th^ day at a specific weather condition relative to a reference value (25^th^ percentile) in all previous 14 days, except for rainfall showing the cumulative relative risk of moderate rainfall versus no rainfall. Males were more susceptible to temperature, humidity and solar radiation, whereas higher wind speed was more influential among females. Both higher humidity and solar radiation had a significantly stronger effect on children aged 3–14 years relative to younger children.

**Table 3:** Stratified cumulative relative risk^a^ of hand, foot and mouth disease hospitalization as a function of meteorological variables for a time lag of 2 weeks.

	Temperature	Relative humidity	Solar radiation	Wind speed	Rainfall
Sex					
Male	0.14* (0.02,0.82)	6.15* (1.05,35.86)	9.96* (3.29,30.16)	1.93 (0.83,4.48)	4.81* (2.14,10.81)
Female	0.25 (0.04,1.70)	3.52 (0.46,26.93)	1.70 (0.48,5.99)	3.32* (1.25,8.86)	2.33 (0.91,5.98)
Age					
0–2	0.30 (0.07,1.32)	1.83 (0.41,8.20)	2.47 (0.97,6.32)	1.79 (0.87,3.69)	2.13* (1.05,4.34)
3–14	0.15 (0.01,2.23)	31.12* (3.16,306.36)	9.00* (2.06,39.21)	2.83 (0.93,8.60)	2.81 (0.95,8.32)

*Significant results

^a^For all meteorological variables, the range for comparison in Table 2 was used for illustration (11°C vs. 19°C was chosen for temperature).

## Discussion

This current study found that, after adjustment for seasonality and long term trend, higher daily mean temperatures, relative humidity, and solar radiation, and moderate levels of wind speed and rainfall were all found to be positively associated with more HFMD cases over a lag of 1–2 weeks.

The exact mechanism behind the relationship between short-term weather variation and HFMD remains unsure. Several proposed factors, including pathogen infectivity outside the host, human behavior, and immune function fluctuations, are involved in the seasonal patterns of infectious diseases [31], and these factors could also be responsible for the relationship between meteorological variations and HFMD. Our findings are generally in line with studies from different regions of Asia [15–17, 21–28]. Our study found a positive association between temperature and HFMD, with increasing trends for mean temperatures between 8°C and 20°C and above 25°C and a plateau between 20°C and 25°C. Similar results have been reported from Rizhao, China, although the plateau for Rizhao was between 10°C and 20°C. [27]. This discrepancy is possibly due to climatic differences between these two cities, given that Rizhao has a temperate, four-season climate. A positive association with temperature has also been reported in Singapore [15], Japan [16], Hong Kong [22] and Zhengzhou, China [28]. There is virological evidence verifying the temperature-sensitive nature of enteroviruses [32] and other human enteric viruses [33]. In addition, warmer weather usually facilitates more outdoor activities, and thus could intensify HFMD transmission through increasing close contacts between individuals.

A hockey-stick effect was found for relative humidity, with significantly increasing risks over 80%, but no strong association below 80%. A positive relationship between humidity and HFMD has been found consistently in Japan, Hong Kong and Rizhao, Guangzhou, and Shenzhen in China [16, 22, 23, 25–27]. Laboratory evidence has shown that enterovirus 70 survival is proportional to the RH level at both moderate and high temperatures (20°C and 33°C) [34]. Fecal-oral has been postulated to be the dominant transmission route for HFMD in developing countries, whereas respiratory pathways could be more important in industrialized countries, due to better personal hygiene and sanitation facilities [35]. Enteroviruses are favored by higher humidity which allows it to persist longer on inanimate surfaces [36].

There could be additional potential delays for rainfall relative to other meteorological variables. Precipitation could have an adverse impact on water and food sanitation, and therefore on HFMD transmission. Moderate rainfall (50^th^–90^th^ percentile, 0 mm<rainfall<17.7 mm) was observed to be associated with an over two-fold higher risk for HFMD hospitalization relative to no rainfall. This is consistent with the finding of a study from Singapore, which reported that moderate precipitation below 75 mm was positively associated with HFMD relative to no rainfall, and that the association reversed when weekly cumulative rainfall was above 75 mm [15]. However, two studies from China found greater rainfall was consistently associated with more HFMD cases [20, 23]. Ground water could be the ideal natural reservoir for maturation and incubation of enteroviruses before its entry to the host. On the other hand, heavy downpours, which could break this transmission by reshaping reservoirs into water currents, could also serve as a protective factor against HFMD. In addition, evidence has shown that physical activity is negatively associated with rainfall [37]. Therefore, extreme precipitation could assist interrupting the transmission by reducing social contacts.

Wind speed and solar radiation were found to be positively associated with HFMD hospitalizations. A previous study in Hong Kong found higher wind speed was associated with more HFMD cases and the authors speculated that stronger wind advanced the spread of disease through airborne droplets [22]. However, our study found a negative association between HFMD and wind speed for wind speeds above 33.6 km/h. A systematic review on the association between ventilation and infection suggested that higher ventilation rates could decrease infection rates or outbreaks of some airborne diseases [38]. Hence, it was likely that strong natural ventilation could serve as a barrier for the spread of respiratory droplets. To date, little is known about the impact of solar radiation on infectious diseases. A study in Hong Kong found solar radiation was positively associated with all respiratory disease hospitalizations [39]. Some vaccine-preventable infectious diseases, including Japanese encephalitis, yellow fever, influenza, rotavirus infection, hepatitis A virus infection and hepatitis B virus infection, were also found to be differentially influenced by ultraviolet radiation from the sun through modulating vector reproduction [40]. In addition, a preventive effect of indoor solar radiation on HFMD was presumed [21]. One plausible reason is that higher solar radiation could also cause temperatures at ground level in urban areas to be higher than temperatures measured at weather stations. Our findings indicated solar radiation and temperature could act synergistically in ways that need further study in subtropical regions where the duration of high temperature and strong radiation are relatively long throughout the year.

Subgroup analyses by gender and age group found that both factors were effect modifiers of the associations between meteorological factors and HFMD. Notably, children aged 3–14 years old were more sensitive to relative humidity and solar radiation than pre-school-age children, as were males compared to females, which could be possibly due to that males and older children tend to be active outdoors rather than be taken care of at home. Further study is needed for the underlying reasons for this disparity.

The present study provided a better understanding of linkages between severe cases of hand, foot and mouth disease, and climate variability in Hong Kong. Along with human behavioral adaptations, the life cycle of enteroviruses could be influenced, directly or indirectly, by meteorological factors, which could potentially affect both the frequency and intensity of HFMD outbreaks. Despite being not able to fully capture the complex disease dynamics in a causal perspective, our study could be serve as an effective tool for establishing an early warning system, in which short-term weather forecasts and environmental surveillance could be adopted to temporally or spatially identify high risk for HFMD outbreaks and thus to prevent epidemics on a local scale. Although it was almost impossible to make prediction on the disease scale based on studies on non-causal association, surveillance capacity could be intensified and also prevention and control strategy, such as health resources allocation, could be enhanced. Moreover, from a clinical perspective, a physician could be alerted when seeing a severe patient with the corresponding symptoms as well as a hot and humid day (or days before) that admission is necessary for the prevention of transmission to a larger population. In addition, global climate change could have a potential impact on the seasonality and interannual pattern of occurrence of infectious diseases as well as the emergence and evolution of new pathogens. The evidence from our study showed the HFMD epidemiology could be partially affected by long-term global warming, as we found higher temperatures were associated with more hospitalizations.

We assessed the relationship between meteorological factors and HFMD on a daily time scale, which could evaluate real-time and delayed effect accurately, given the incubation period for HFMD and time lags for the effect of weather predictors were relatively short. Furthermore, we modelled the cumulative effect of meteorological variables from the first day until the maximum lags. Cumulative risks are presented as they explicitly account for same day as well as lagged effects, and thus better reflect the true impact of meteorological variables. Notwithstanding, a few limitations of the current study need to be considered. First, we used the hospitalization data for analysis, which likely underestimated the overall incidence in Hong Kong, given that most HFMD patients recover without any clinical attention. However, our study captured the impact of weather variations on more severe HFMD cases that arguably are of more public health significance. Second, the information on separation of pathogens causing HFMD was not available in this study. Because CA16 and EV71 could have distinct yearly seasonality based on region [14], HFMD induced by different types of enteroviruses could be influenced differentially by meteorological factors. Further study is desirable with laboratory-confirmed causes of disease. Third, the values of meteorological variables we used in the analysis were measured at a single central monitoring station in Hong Kong, which may not reflect the actual exposure of the majority in the target population.

## Conclusions

The present study provides quantitative evidence that short-term meteorological variations could be used as early indicators for potential HFMD outbreaks. Climate change is likely to lead to a substantial increase in severe HFMD cases in this subtropical city in the absence of further interventions.

## References

[1] Duff MF. Hand-foot-and-mouth syndrome in humans: coxackie A10 infections in New Zealand. Br Med J. 1968;2(5606):661–4. 5658411 10.1136/bmj.2.5606.661 PMC1991723

[2] Chen KT, Chang HL, Wang ST, Cheng YT, Yang JY. Epidemiologic features of hand-foot-mouth disease and herpangina caused by enterovirus 71 in Taiwan, 1998–2005. Pediatrics. 2007;120(2):e244–52. 17671037 10.1542/peds.2006-3331

[3] Ma E, Lam T, Chan KC, Wong C, Chuang SK. Changing epidemiology of hand, foot, and mouth disease in Hong Kong, 2001–2009. Jpn J Infect Dis. 2010;63(6):422–6. 21099093

[4] Ang LW, Koh BK, Chan KP, Chua LT, James L, Goh KT. Epidemiology and control of hand, foot and mouth disease in Singapore, 2001–2007. Ann Acad Med Singapore. 2009;38(2):106–12. 19271036

[5] Nguyen NT, Pham HV, Hoang CQ, Nguyen TM, Nguyen LT, Phan HC, et al Epidemiological and clinical characteristics of children who died from hand, foot and mouth disease in Vietnam, 2011. BMC Infect Dis. 2014;14:341 10.1186/1471-2334-14-341 24942066 PMC4068316

[6] World Health Organization Western Pacific Region. A Guide to clinical management and public health response for hand, foot and mouth disease (HFMD). 1 ed Geneva: World Health Organization; 2012.

[7] Zhang T, Du J, Xue Y, Su H, Yang F, Jin Q. Epidemics and Frequent Recombination within Species in Outbreaks of Human Enterovirus B-Associated Hand, Foot and Mouth Disease in Shandong China in 2010 and 2011. PLoS One. 2013;8(6):e67157 10.1371/journal.pone.0067157 23840610 PMC3686723

[8] Ventarola D, Bordone L, Silverberg N. Update on hand-foot-and-mouth disease. Clin Dermatol. 2015;33(3):340–6. 10.1016/j.clindermatol.2014.12.011 25889136

[9] World Health Organization Western Pacific Region. Hand, Foot, and Mouth Disease Situation Update. Geneva: 2015.

[10] World Health Organization Western Pacific Region. Hand, Foot, and Mouth Disease Situation Update. Genava: 2014.

[11] Wu Y, Yeo A, Phoon MC, Tan EL, Poh CL, Quak SH, et al The largest outbreak of hand; foot and mouth disease in Singapore in 2008: the role of enterovirus 71 and coxsackievirus A strains. Int J Infect Dis. 2010;14(12):e1076–81. 10.1016/j.ijid.2010.07.006 20952237

[12] Centre for Health Protection. EV Scan 2016 [updated 2016 Jun 10th; cited 2016 Jun 10th]. Available: http://www.chp.gov.hk/en/guideline1_year/29/134/441/502.html.

[13] Blomqvist S, Klemola P, Kaijalainen S, Paananen A, Simonen ML, Vuorinen T, et al Co-circulation of coxsackieviruses A6 and A10 in hand, foot and mouth disease outbreak in Finland. J Clin Virol. 2010;48(1):49–54. 10.1016/j.jcv.2010.02.002 20189452

[14] Chua KB, Kasri AR. Hand foot and mouth disease due to enterovirus 71 in Malaysia. Virol Sin. 2011;26(4):221–8. 10.1007/s12250-011-3195-8 21847753 PMC8222466

[15] Hii YL, Rocklov J, Ng N. Short term effects of weather on hand, foot and mouth disease. PLoS One. 2011;6(2):e16796 10.1371/journal.pone.0016796 21347303 PMC3037951

[16] Onozuka D, Hashizume M. The influence of temperature and humidity on the incidence of hand, foot, and mouth disease in Japan. Sci Total Environ. 2011;410–411:119–25. 10.1016/j.scitotenv.2011.09.055 22014509

[17] Li T, Yang Z, Di B, Wang M. Hand-foot-and-mouth disease and weather factors in Guangzhou, southern China. Epidemiol Infect. 2014;142(8):1741–50. 10.1017/S0950268813002938 24267476 PMC9151230

[18] Fan J, Lin H, Wang C, Bai L, Yang S, Chu C, et al Identifying the high-risk areas and associated meteorological factors of dengue transmission in Guangdong Province, China from 2005 to 2011. Epidemiol Infect. 2014;142(3):634–43. 10.1017/S0950268813001519 23823182 PMC9161228

[19] Chong KC, Goggins W, Zee BC, Wang MH. Identifying meteorological drivers for the seasonal variations of influenza infections in a subtropical city—Hong Kong. Int J Environ Res Public Health. 2015;12(2):1560–76. 10.3390/ijerph120201560 25635916 PMC4344680

[20] Cheng J, Wu J, Xu Z, Zhu R, Wang X, Li K, et al Associations between extreme precipitation and childhood hand, foot and mouth disease in urban and rural areas in Hefei, China. Sci Total Environ. 2014;497–498:484–90. 10.1016/j.scitotenv.2014.08.006 25150743

[21] Wu X, Sun Y, Lin C, Jia L, Wu Q, Li X, et al A case-control study to identify environmental risk factors for hand, foot, and mouth disease outbreaks in Beijing. Jpn J Infect Dis. 2014;67(2):95–9. 24647250 10.7883/yoken.67.95

[22] Ma E, Lam T, Wong C, Chuang SK. Is hand, foot and mouth disease associated with meteorological parameters? Epidemiol Infect. 2010;138(12):1779–88. 10.1017/S0950268810002256 20875200

[23] Wang Y, Feng Z, Yang Y, Self S, Gao Y, Longini IM, et al Hand, foot, and mouth disease in China: patterns of spread and transmissibility. Epidemiology. 2011;22(6):781–92. 10.1097/EDE.0b013e318231d67a 21968769 PMC3246273

[24] Zou XN, Zhang XZ, Wang B, Qiu YT. Etiologic and epidemiologic analysis of hand, foot, and mouth disease in Guangzhou city: a review of 4,753 cases. Braz J Infect Dis. 2012;16(5):457–65. 10.1016/j.bjid.2012.08.001 22964289

[25] Lin H, Zou H, Wang Q, Liu C, Lang L, Hou X, et al Short-term effect of El Nino-Southern Oscillation on pediatric hand, foot and mouth disease in Shenzhen, China. PLoS One. 2013;8(7):e65585 10.1371/journal.pone.0065585 23935817 PMC3720731

[26] Chen C, Lin H, Li X, Lang L, Xiao X, Ding P, et al Short-term effects of meteorological factors on children hand, foot and mouth disease in Guangzhou, China. Int J Biometeorol. 2014;58(7):1605–14. 10.1007/s00484-013-0764-6 24258319

[27] Wu H, Wang H, Wang Q, Xin Q, Lin H. The effect of meteorological factors on adolescent hand, foot, and mouth disease and associated effect modifiers. Glob Health Action. 2014;7:24664 10.3402/gha.v7.24664 25098727 PMC4124175

[28] Feng H, Duan G, Zhang R, Zhang W. Time series analysis of hand-foot-mouth disease hospitalization in Zhengzhou: establishment of forecasting models using climate variables as predictors. PLoS One. 2014;9(1):e87916 10.1371/journal.pone.0087916 24498221 PMC3909295

[29] Gasparrini A. Distributed Lag Linear and Non-Linear Models in R: The Package dlnm. J Stat Softw. 2011;43(8):1–20. 22003319 PMC3191524

[30] Wood S. Generalized Additive Models: An Introduction with R. 1 ed Boca Raton: Chapman and Hall/CRC; 2006.

[31] Fares A. Factors influencing the seasonal patterns of infectious diseases. Int J Prev Med. 2013;4(2):128–32. 23543865 PMC3604842

[32] Kung YH, Huang SW, Kuo PH, Kiang D, Ho MS, Liu CC, et al Introduction of a strong temperature-sensitive phenotype into enterovirus 71 by altering an amino acid of virus 3D polymerase. Virology. 2010;396(1):1–9. 10.1016/j.virol.2009.10.017 19906393

[33] Rzezutka A, Cook N. Survival of human enteric viruses in the environment and food. FEMS Microbiol Rev. 2004;28(4):441–53. 15374660 10.1016/j.femsre.2004.02.001

[34] Sattar SA, Dimock KD, Ansari SA, Springthorpe VS. Spread of acute hemorrhagic conjunctivitis due to enterovirus-70: effect of air temperature and relative humidity on virus survival on fomites. J Med Virol. 1988;25(3):289–96. 2844979 10.1002/jmv.1890250306

[35] Wong SS, Yip CC, Lau SK, Yuen KY. Human enterovirus 71 and hand, foot and mouth disease. Epidemiol Infect. 2010;138(8):1071–89. 10.1017/S0950268809991555 20056019

[36] Kramer A, Schwebke I, Kampf G. How long do nosocomial pathogens persist on inanimate surfaces? A systematic review. BMC Infect Dis. 2006;6:130 16914034 10.1186/1471-2334-6-130 PMC1564025

[37] Belanger M, Gray-Donald K, O'Loughlin J, Paradis G, Hanley J. Influence of weather conditions and season on physical activity in adolescents. Ann Epidemiol. 2009;19(3):180–6. 10.1016/j.annepidem.2008.12.008 19217000

[38] Atkinson J, Chartier Y, Pessoa-Silva CL, Jensen P, Li Y, Seto W-H. Natural Ventilation for Infection Control in Health-Care Settings. 1 ed Geneva: World Health Organization; 2010. 23762969

[39] Chan EY, Goggins WB, Yue JS, Lee P. Hospital admissions as a function of temperature, other weather phenomena and pollution levels in an urban setting in China. Bull World Health Organ. 2013;91(8):576–84. 10.2471/BLT.12.113035 23940405 PMC3738307

[40] Guo B, Naish S, Hu W, Tong S. The potential impact of climate change and ultraviolet radiation on vaccine-preventable infectious diseases and immunization service delivery system. Expert Rev Vaccines. 2015;14(4):561–77. 10.1586/14760584.2014.990387 25493706

